# Cinnamic Acid and Its Derivatives: Mechanisms for Prevention and Management of Diabetes and Its Complications

**DOI:** 10.3390/nu9020163

**Published:** 2017-02-21

**Authors:** Sirichai Adisakwattana

**Affiliations:** Department of Nutrition and Dietetics, Faculty of Allied Health Sciences, Chulalongkorn University, Bangkok 10330, Thailand; Sirichai.a@chula.ac.th; Tel.: +66-2-218-1099 (ext. 111)

**Keywords:** cinnamic acid and its derivatives, mechanisms, diabetes, complications

## Abstract

With recent insight into the development of dietary supplements and functional foods, search of effective phytochemical compounds and their mechanisms involved in prevention and management of diabetes and its complications are now being assessed. Cinnamic acid and its derivatives occur naturally in high levels of plant-based foods. Among various biological activities, cinnamic acid and its derivatives are associated with a beneficial influence on diabetes and its complications. The aim of the review is to summarize the potential mechanisms of these compounds for prevention and management of diabetes and its complications. Based on several in vitro studies and animal models, cinnamic acid and its derivatives act on different mechanism of actions, including stimulation of insulin secretion, improvement of pancreatic β-cell functionality, inhibition of hepatic gluconeogenesis, enhanced glucose uptake, increased insulin signaling pathway, delay of carbohydrate digestion and glucose absorption, and inhibition of protein glycation and insulin fibrillation. However, due to the limited intestinal absorption being a result of low bioavailability of cinnamic acid and its derivatives, current improvement efforts with entrapping into solid and liquid particles are highlighted. Further human clinical studies are needed to clarify the effects of cinnamic acid and its derivatives in diabetic patients.

## 1. Introduction

Type 2 diabetes is a group of metabolic disorder characterized by hyperglycemia dyslipidemia, and protein metabolism due to insulin resistance, impaired insulin signaling, and β-cell dysfunction. The prevalence of type 2 diabetes mellitus has increased dramatically in epidemic proportions worldwide that is one of the most important health and socioeconomic problems [1]. Long-term hyperglycemia causes the development and progression of pathogenic conditions including micro- and macro-vascular complications, neuropathy, retinopathy, nephropathy, and a consequent reduction in quality of life and an increase in the risk of mortality and morbidity [2]. Nowadays, the general guidelines for treatment and management of type 2 diabetes mellitus recommend dietary modification including other aspects of lifestyle modification by increased the quantity and quality of physical activity, along with pharmacological interventions [3].

Current anti-diabetic drugs are commonly required for treatment of type 2 diabetes aimed to achieve glycemic control and relieve diabetic symptoms. Currently, there are six distinct classes of available hypoglycemic agents: sulfonylureas, meglitinides, biguanides, thiazolidinediones, α-glucosidase inhibitors, and dipeptidyl peptidase-IV (DPP-IV) inhibitors [4]. Each drug class displays unique mechanisms of action, including stimulation of insulin secretion, inhibition of hepatic gluconeogenesis, increased insulin receptor sensitivity and delay of carbohydrate digestion, respectively. Inhibitors of sodium-glucose cotransporters type 2 (SGLT2) are a new oral hypoglycemic agent by inhibiting reabsorption of glucose in proximal convoluted tubule [5]. According to the statement by the American Diabetes Association (ADA) and the European Association for the study of Diabetes (EASD), SGLT2 inhibitors are currently integrated as second- or third-line therapy for the treatment of type 2 diabetes mellitus [6]. However, oral hypoglycemic agents could produce severe hypoglycemia, weight gain, and gastrointestinal disturbances. In the last decade, dietary polyphenols have received much attention from scientists, researchers, and food manufacturers as new promising agents for prevention and management of diabetes and its complications because they are abundant in nature, inexpensive to produce and may have less side effects than currently used hypoglycemic agents [7]. Cinnamic acid and its derivatives are the major group of phenolic acids with ubiquitous distribution in fruits and vegetables. Recent data support their beneficial effects, including antioxidant [8], anti-inflammatory [9], and anti-cancer activities [10]. Some of them are effective in reduction of blood glucose levels in animal models [11]. Anti-diabetic mechanisms underlying how cinnamic acid and its derivatives lower blood glucose levels have been continuously studied. Therefore, the present review aims to summarize recent literatures linking the effect of cinnamic acid and its derivatives on prevention and management of diabetes and its complications and to describe the multiple mechanisms of action, which were based on evidence from laboratory experiments and animal models.

## 2. Dietary Sources of Cinnamic Acid and Its Derivatives

**Figure 1:**
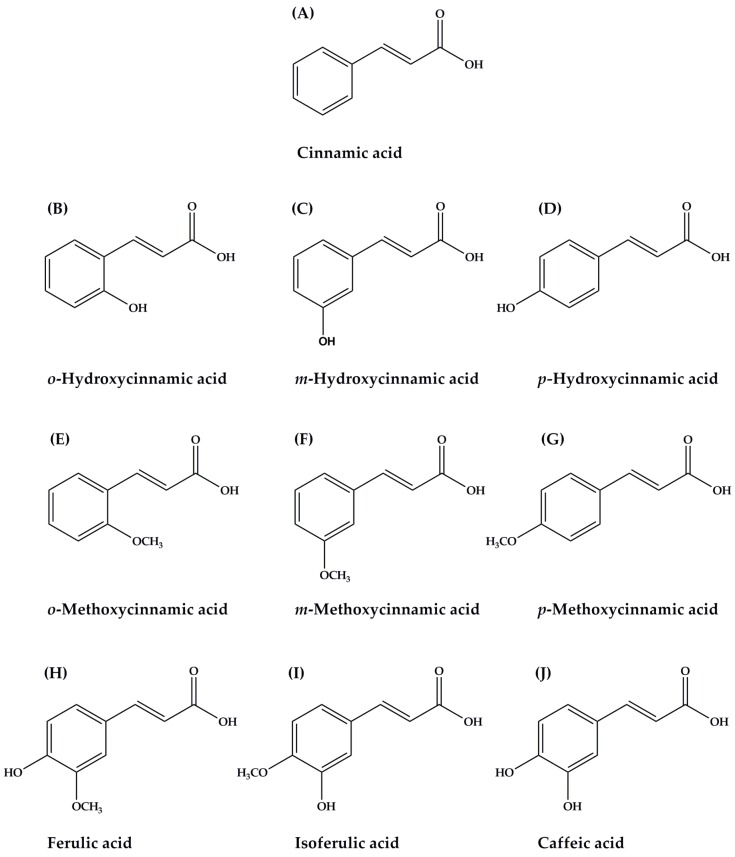
The chemical structure of cinnamic acid and its derivatives. (**A**) Cinnamic acid; (**B**) *o*-Hydroxycinnamic acid; (**C**) *m*- Hydroxycinnamic acid; (**D**) *p*- Hydroxycinnamic acid; (**E**) *o*-Methoxycinnamic acid; (**F**) *m*-Methoxycinnamic acid; (**G**) *p*-Methoxycinnamic acid; (**H**) Ferulic acid (**I**) Isoferulic acid; (**J**) Caffeic acid.

Polyphenols are one of the most important and abundant phytochemical compounds in human diets. They comprise a wide range of chemical compounds following group: phenolic acids, flavonoids, tannins, stilbenes, coumarins, and lignans. Phenolic acids are usually classified into two major groups: benzoic acids, containing seven carbon atoms (C6-C1), and cinnamic acids, comprising nine carbon atoms (C6-C3). Cinnamic acid and its derivatives (Figure 1), naturally occurring bioactive compounds, are synthesized in the plants by following the shikimate pathway where phenylalanine and tyrosine are two precursor molecules [12,13]. Biosynthetic pathway of cinnamic acids leads to the synthesis of various phytochemical compounds such as coumarins, lignans, flavonoids, stilbenes, anthocyanins, and tannins [14]. Among the most common and well-known cinnamic acid and its derivatives are cinnamic acid (Figure 1A), caffeic acid (Figure 1J), ferulic acid (Figure 1H), isoferulic acid (Figure 1I), and *p*-hydroxycinnamic acid (Figure 1D). The variety of cinnamic acid and its derivatives are abundantly found in plant-based foods such as fruits, vegetables, and whole grains. For instance, ferulic acid is the most predominant in many plants such as cereal grains, rice, and wheat bran [13]. Coffee is the primary source of caffeic acid in the human diet [15]. Other edible plants that have been found to contain caffeic acid include sweet potatoes (*Ipomoea batatas* L.) [16] and artichoke (*Cynara cardunculus* L.) [17]. In addition, cinnamic acid can be generally obtained from cinnamon (*Cinnamomum cassia* (L.) J.Presl), citrus fruits, grape (*Vitis vinifera* L.), tea (*Camellia sinensis* (L.) Kuntze), cocoa (*Theobroma cacao* L.), spinach (*Spinacia oleracea* L.), celery (*Apium graveolens* L.), and brassicas vegetables [18]. Isoferulic acid is commonly found in Chinese propolis [19] and Cimicifuga (*Cimicifuga heracleifolia* var. *bifida* Nakai), an herbal medicine in oriental countries such as Japan and China [20]. Moreover, the most important dietary resources of *p*-hydroxycinnamic acid (*p*-coumaric acid) are peanuts (*Arachis hypogaea* L.) [21], basil (*Ocimum basilicum* L.) [22], and garlic (*Allium sativum* L.) [23]. *p*-Methoxycinnamic acid is a component of medicinal plants such as Granny’s Nightcap (*Aquilegia vulgaris* L.) [24] and Buerger’s Figwort (*Scrophularia buergeriana* Miq.) [25].

As they occur widely and abundantly in human diets, several studies have provided evidence of daily consumption of cinnamic acid and its derivatives in worldwide populations. A German study estimated daily consumption of hydroxycinnamic acids at 211 mg/day and the principal sources were coffee for caffeic acid and fruit and fruit juices for *p*-hydroxycinnamic acid [26]. A Polish study assessed the estimated hydroxycinnamic acids intake, which was 150 mg/day that largely originated from coffee [27]. The French cohort SUpplémentation en VItamines et Minéraux AntioXydants (SU.VI.MAX) reported that estimated mean intakes of hydroxycinnamic acids was 599 mg/day and 316 mg as aglycones/day [28]. The European Prospective Investigation into Cancer and Nutrition (EPIC) study revealed that the most frequently consumed hydroxycinnamic acids were caffeic acid (188.6–626.2 mg/day), ferulic acid (45.0–159.3 mg/day) and *p*-hydroxycinnamic acid (11.7–17.9 mg/day) in all European regions [29]. The main food sources of hydroxycinnamic acids intake were from coffee and other food sources were fruits, nuts and seeds, some vegetables, and cereal and cereal products. Pharmacokinetic studies have shown that cinnamic acid and its derivatives are absorbed easily from the small intestine through various mechanisms such as passive diffusion [30,31], monocarboxylic acid transporters (MCTs) [31,32], and carrier-mediated transport with involvement of a Na^+^-dependent [33].

## 3. Potential Mechanisms of Action of Cinnamic Acid and Its Derivatives in Diabetes and Its Complications

**Figure 2:**
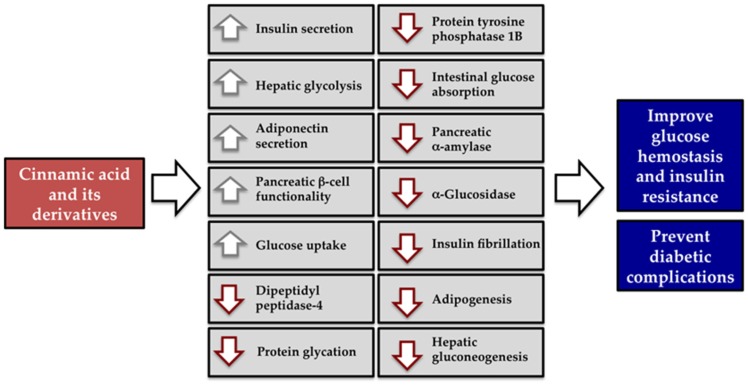
Schematic diagram represents the mechanism actions of cinnamic acid and its derivatives for prevention and management of diabetes and its complication. (↑) Increase, (↓) Decrease.

Cinnamic acid and its derivatives have received increasing attention in recent years due to the high number of beneficial health properties attributed to their consumption. In this review, the effects of cinnamic acid and its derivatives (Figure 1) are discussed with particular insight into the underlying mechanisms on prevention and management of diabetes and its complications. As summarized in Figure 2, several mechanisms have been proposed to explain the beneficial effects of cinnamic acid and its derivatives related to diabetes and its complications.

### 3.1. Insulin Secretion

Elevated circulating concentration of glucose is mainly regulated by insulin secretion from pancreatic β-cells. The stimulatory insulin secretion from β-cells occurs when glucose enters the cells through glucose transporter 2 (GLUT2) and is then phosphorylated to glucose-6 phosphate by glucokinase and then metabolized via glycolysis and oxidation. The generation of ATP by glycolysis, the Krebs cycle and the respiratory chain induces the closure of the ATP-sensitive K^+^ channels (KATP channels), which evokes membrane depolarization and subsequently activates and opens the voltage-dependent Ca^2+^ channels (VDCCs) [34]. This effect causes an increase in [Ca^2+^]_i_, then the stimulatory insulin release from the vesicles to plasma membrane. Several studies have addressed the potential stimulatory insulin secretion of cinnamic acid and its derivatives using cell lines and animal models. Screening insulin-secreting activity of cinnamic acid and its derivatives has been studied in INS-1 pancreatic β-cells [35]. In acute exposure experiments, the INS-1 pancreatic β-cells were incubated with nine cinnamic acid and its derivatives. According to a structure-activity relationship analysis, the key pharmacophore to stimulate insulin secretion was the single introduction of hydroxy-substituted groups at meta-position or methoxy-substituted groups at para-position on cinnamic acid [35]. The structure of cinnamic acid was then substituted with two substituted groups following this evidence, resulting in isoferulic acid (3-hydroxy-4-methoxycinnamic acid) [35]. It was surprising that isoferulic acid had no effect on insulin secretion. In contrast, the presence of para-hydroxy and meta-methoxy groups in cinnamic acid structure (ferulic acid) exhibited the highest insulin secreting activity among the various cinnamic acid derivatives tested [35]. This stimulated insulin secretory response of ferulic acid was confirmed by using the pancreatic rat perfusion technique [35]. The onset time of insulin secretion stimulated by ferulic acid (100 μM) was less than 1 min and reached the peak at 4 min that was about 3.4-fold of the baseline level. Ferulic acid (5 mg/kg) produced a marked increase in plasma insulin concentration with concomitant decline in plasma glucose concentration in normal rats after 6 min of intravenous injection [35].

In a similar study, *p*-methoxycinnamic acid (10–100 μM) stimulated insulin release at the basal glucose level, while it (10 μM) also enhanced glucose-induced insulin secretion in the perfused rat pancreas and INS-1 pancreatic β-cells [36]. At the same concentration, *p*-methoxycinnamic acid could induce an increase in [Ca^2+^]_i_ in INS-1 pancreatic β-cells [36]. This suggests that the mechanism of stimulatory insulin secretion by *p*-methoxycinnamic acid is due to an increasing in Ca^2+^ influx through the l-type Ca^2+^ channels without induction of membrane depolarization by closing of K_ATP_ channels [37]. Two possible mechanisms of action have been proposed that *p*-methoxycinnamic acid may induce membrane depolarization via K_ATP_-independent pathways (chloride-channel and sodium-permeable cation channel) and activate cAMP-dependent mechanisms that modulate the calcium influx through l-type Ca^2+^ channel [37]. *p*-Methoxycinnamic acid (10 μM) also enhanced the insulin secreting activity of glibenclamide-induced insulin secretion in pancreatic β-cells. In this view, the insulin secreting activity of *p*-methoxycinnamic acid has been extensively studied in both normal and streptozotocin (STZ)-induced diabetic rats [36]. In both fasting and glucose loading conditions, *p*-methoxycinnamic acid (10–100 mg/kg) lowered plasma glucose concentration with concomitant increased plasma insulin concentration in both normal and STZ-induced diabetic rats [36]. Interestingly, *p*-methoxycinnamic acid decreased fasting plasma glucose and elevated fasting plasma insulin concentrations at doses of 40 mg/kg without severe reduction of plasma glucose in normal rats. The overall findings suggest that *p*-methoxycinnamic acid improves glucose tolerance without hypoglycemia, which may be beneficial to diabetic conditions that have defects in the response of insulin secretion to glucose stimulation.

Cinnamic acid (100 μM) was found to be an inactive insulin-secreting compound in INS-1 pancreatic β-cells [35]. In consistent with the isolated mice islets, cinnamic acid had no effect on insulin secretion at the basal glucose level (3 mM), however, it (50–200 μM) enhanced glucose-induced insulin secretion in a concentration-dependent manner under high glucose concentration (16 mM) [38]. Anti-diabetic activity of cinnamic acid was confirmed using oral glucose tolerance test. Oral administration of cinnamic acid (5 and 10 mg/kg) markedly improved glucose tolerance in diabetic rats [38]. These findings suggest that cinnamic acid exerts anti-diabetic activity by improving glucose tolerance and insulin secretion. Caffeic acid (0.1 nM–1 μM) had no insulin secreting activity at 3.3 mM glucose, but it enhanced 16.7 mM glucose-induced insulin secretion in INS-1E pancreatic β-cells [39]. This enhancement of caffeic acid might improve glucose-induced insulin secretion in the condition of diabetes. The effects of cinnamic acid and its derivatives on stimulatory insulin secretion are summarized in Table 1.

### 3.2. Pancreatic β-Cell Functionality

Chronic exposure to hyperglycemia can lead to glucotoxicity that pertains to the dysfunction of pancreatic β-cell characterized by decreasing insulin gene expression with and accelerated apoptosis [40]. The responsible metabolic lesion appears to involve a defect in insulin synthesis and secretion. Impaired pancreatic β-cell function plays a pivotal role in the development of type 1 and type 2 diabetes. Bhattacharya et al. studied the protective effect of caffeic acid against glucose-induced toxicity in pancreatic β-cells [39]. An increase in caspase-3-dependent beta-cell apoptosis and downregulation of anti-apoptotic gene Bax (belonging to the Bcl-2 family) was observed under the condition of 25 mM glucose for 72 h [39]. The alteration in apoptotic gene expression was attenuated by caffeic acid (0.1 nM–1 μM). Caffeic acid markedly increased the gene expression of the key β-cell function (Glut2 (glucose transporter 2), Ins1 (insulin 1), Ins2, Beta2 (neurogenic differentiation protein 1), Pdx1 (pancreatic and duodenal homeobox protein 1), Akt1 (RAC-α serine/threonine-protein kinase encoding gene), and Akt2 (RAC-β serine/threonine-protein kinase encoding gene)) [39]. They suggest that caffeic acid produced cytoprotective effect and improved β-cell survival and function through increased expression of key β-cell survival and regulatory genes during glucotoxicity. Caffeic acid phenethyl ester (CAPE), the ester of caffeic acid and phenethyl alcohol, is one of the major components of honeybee propolis. CAPE (5 μM/kg intraperitoneal injection/every two days) has been shown a significant glucose-lowering effect on type 1 diabetic mice with the reduced inflammation through a decline in level of IL-1β and IFN-γ [41]. Histopathological determination indicated that destroyed pancreatic islets were regenerated after treatment of CAPE. The authors suggest that anti-diabetic effect may be related to its anti-inflammatory and angiostatic effects.

Ferulic acid has remarkable effects against STZ-induced pancreatic β-cell damage [42]. Roy et al. demonstrated that treatment with ferulic acid (50 mg/kg daily) to STZ-induced diabetic rats reduced blood glucose concentration after 8 weeks [42]. Histological appearance indicated an increase in the number of pancreatic islets and the number of β-cells, along with a reduction in the number of vacuolation [42]. In consistent with reduction in the activity of apoptosis, ferulic acid also suppressed β-cell inflammation by modulating expression of transforming growth factor (TGF-β1) and interleukin-1β (IL-1β) [42]. Streptozotocin (STZ) has been long used for induction of diabetes in animal models. STZ is transported into the pancreatic β-cell through a glucose transporter-2 (GLUT2) that causes alkylation of DNA and generates hydrogen peroxide and hydroxyl radicals leading to an inflammatory stress and β-cell dysfunction and death [43]. It is suggested that ferulic acid might act as an antioxidant to help neutralize the harmful effects of free radicals generated by STZ, leading to reduce inflammation and apoptosis in pancreatic β-cell. Prabhakar et al. demonstrated the effect of ferulic acid and co-administered with oral hypoglycemic agents in STZ-induced diabetic rats [44]. Ferulic acid (10 and 40 mg/kg) exhibited synergistic effect with metformin and thiazolidinedione on the improvement of blood glucose and lipid profile in STZ-induced diabetic rats after three week of administration. Synergistic actions by increased islet number and sizes and reduced insulitis grades near to normal values were observed when co-administration of ferulic acid and metformin or thiazolidinedione. The increased β-cell mass improved the ability to secrete insulin which might increase the peripheral utilization of glucose [45]. One possible mechanism of ferulic acid for preventing the loss of islet numbers and sizes is through free radical scavenging activity. Prabhakar et al. suggest that synergistic interaction of ferulic acid and oral hypoglycemic drugs could provide a prospect to reduce the dose of oral hypoglycemic drugs that may help in diminishing their adverse effects as well as achieve enhanced therapeutic actions [44].

Palmitic acid is known as an inducer for the lipid accumulation and lipotoxicity, leading to accelerated apoptosis and loss of function and mass in pancreatic β-cells [46]. It was described that palmitic acid causes a remarkable decrease in adenosine monophosphate-activated protein kinase (AMPK) protein expression and its downstream targets such as phosphoacetyl-coA carboxylase (pACC) and carnitine acyl transferase 1 (CPT-1). Consequently, the downstream effect of suppressing AMPK increases lipogenic gene and protein expression including SREBP-1c mRNA, fatty acid synthase (FAS), and ACC, resulting in the lipid accumulation in pancreatic β-cells [46]. Very recently, the effect of cinnamic acid on palmitic acid-induced intracellular triglyceride accumulation has been tested in mouse NIT-1 pancreatic β-cells [47]. Cinnamic acid (100 μM) inhibited palmitic acid-induced alteration of lipogenic gene and protein expression (AMPK, FAS, ACC, and CPT-1) without significant reduction in intracellular triglyceride content in pancreatic β-cells [47]. The effects of cinnamic acid and its derivatives on pancreatic β-cell functionality are summarized in Table 1.

### 3.3. Dipeptidyl Peptidase-IV (DPP-IV)

Inhibition of dipeptidyl peptidase-IV (DPP-IV) is a new therapeutic approach for management of type 2 diabetes [48]. DPP-IV inhibitors, such as sitagliptin and saxagliptin, slow the inactivation and degradation of glucagon-like peptide-1 (GLP-1) that stimulates the secretion of insulin from pancreatic β-cells and decreases the secretion of glucagon from pancreatic α-cells [48]. Adolpho et al. described the preliminary screening of cinnamic acid and its derivatives against DPP-IV [49]. At concentration of 500 μM, cinnamic acid, *p*-methoxycinnamic acid and caffeic acid showed inhibitory activity with 4.4%, 11.5% and 50.1%, respectively [49]. More recently, Fan et al. found that caffeic acid was able to inhibit DPP-IV with the IC_50_ value of 3.37 μM, which was the same potency as a positive control (IC_50_ = 4.21 μM) [50]. Computational docking analysis revealed that hydrogen bonding and the formation of π-interaction were the main binding mode of caffeic acid with DPP-IV [50]. The binding of caffeic acid caused DPP-IV conformational changes, or changes in the side chain of amino acid residues of DPP-IV, leading to decrease its binding ability to the substrate. The effects of cinnamic acid and its derivatives on inhibition of DPP-IV are summarized in Table 2.

### 3.4. Glucose Uptake

It is now well established that adipose tissue and skeletal muscle are main targets of insulin-stimulated glucose uptake to control of hyperglycemia. In skeletal muscle and adipose tissues, insulin-stimulated glucose uptake primarily occurs through several signaling pathways. Upon binding to its receptor, insulin activates glucose uptake through a distinct signaling cascade involving multiple enzymes of which the proteins phosphoinositide 3-kinase (PI3K), the Akt/PKB, and the PKCζ cascades, thereby resulting in the translocation of GLUT4 to plasma membrane [51]. In adipocytes, peroxisome proliferator activated receptor γ (PPARγ) activation increases the expression and translocation of GLUT4 to the cell membrane, thus increasing the uptake of glucose, and reducing subsequently plasma glucose levels [52]. There are interesting studies, which have addressed the stimulating effects of cinnamic acid and its derivatives on glucose uptake in C_2_C_12_ myoblast cells [53]. It was noted that cinnamic acid lacked the ability to stimulate glucose uptake, while ferulic acid and isoferulic acid resulted in a smaller increase in glucose uptake into C_2_C_12_ cells [53]. The authors suggest that the introduction of hydroxyl group at the meta- and/or para-position on the benzene ring of cinnamic acid raises the ability to stimulate glucose uptake into the cells [53]. However, the stimulating effect disappeared when the hydroxy groups on the benzene ring of cinnamic acid were totally substituted for methoxy groups. In addition, cinnamic acid derivatives enhance the uptake of glucose into C_2_C_12_ cells via the activation of α-1A adrenoceptor subtype. The additional study revealed that the activation of α-1A adrenoceptor by isoferulic acid (0.1 nM–1 mM) increases the glucose uptake via PLC-PKC pathway in C_2_C_12_ cells [54].

**Figure 3:**
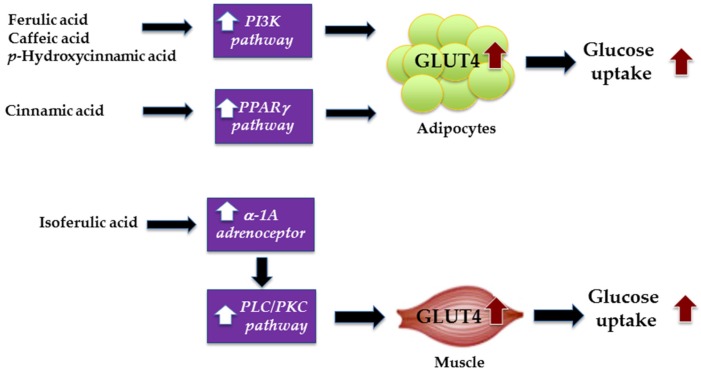
Mechanisms of ferulic acid, caffeic acid, *p*-hydroxycinnamic acid, cinnamic acid, and isoferulic acid for stimulating glucose uptake in adipocytes and muscle.

The effect of cinnamic acid and its derivatives (cinnamic acid, ferulic acid, *p*-hydroxycinnamic acid, and caffeic acid) and their combinations with oral hypoglycemic drugs (metformin and thiazolidinedione) has been described to promote synergistic effect in the glucose uptake activity in 3T3-L1 adipocytes [55]. Prabhakar et al. found that tested compounds showed different signaling mechanisms on the increased glucose uptake. Ferulic acid (25 μM), caffeic acid (25 μM), and *p*-hydroxycinnamic acid (25 μM) stimulated the glucose uptake mediated by PI3K-dependent GLUT4 translocation, whereas cinnamic acid increased the glucose uptake via PPARγ-mediated GLUT4 translocation in mature 3T3-L1 adipocytes [55]. In combination with oral hypoglycemic agents, ferulic acid, caffeic acid, and *p*-hydroxycinnamic acid synergistically interacted with metformin (20 μM) and thiazolidinedione (20 μM), whereas cinnamic acid exhibited an additive effect on the uptake of glucose. The synergistic effect was also observed in rat L6 myotubes when combination of ferulic acid with metformin or thiazolidinedione [44]. The increase in mRNA expression of PI3K was detected when L6 myotubes were treated with ferulic acid. The authors suggest that the combination therapy between compounds and oral hypoglycemic agents may enhance the beneficiary activities and reduce the drug load to the patients. The mechanisms of ferulic acid, caffeic acid, *p*-hydroxycinnamic acid, cinnamic acid, and isoferulic acid for stimulating glucose uptake in adipocytes and muscle are illustrated in Figure 3.

Saturated fatty acid has been reported to induce impaired insulin sensitivity in muscle by induction of phosphorylation of PKCε [56]. This leads to defects in high mobility group A1 (HMGA1) protein, leading to downregulation of insulin receptor β mRNA expression. Impairment in insulin receptor expression directly alters insulin-stimulated GLUT4 translocation to the plasma membrane. Gogoi et al. described that ferulic acid (2–20 μg/mL) prevented saturated fatty acid-induced defects in the insulin receptor through the blockage of PKCε activation and thereby permission of HMGA1 to activate insulin receptor β promoter in rat L6 myotubes [57]. Consistent with ferulic acid (0.6 mg/kg body weight/day), improved glycemic level in insulin-resistant high-fat diet (HFD) rat model occurred within 15 days when orally administered for eight days. It suggests that ferulic acid attenuates the saturated fatty acid-induced defects in the insulin receptor, which is responsible for improvement of insulin resistance in the muscle. The effects of cinnamic acid and its derivatives on the glucose uptake are summarized in Table 2.

### 3.5. Hepatic Glucose Homeostasis

The liver plays a key role for the maintenance of blood glucose homeostasis in normal and diabetic states. Targeting key metabolic and regulatory hepatic enzymes to enhance glycolysis and suppress gluconeogenesis serve as feasible approaches for treatment and management of diabetes [58]. There are interesting studies demonstrating the modulatory effects of cinnamic acid and its derivatives on many aspects of gene and protein expression related to cellular signaling transduction of insulin-targeting organs [59,60,61].

Cinnamic acid derivatives are able to regulate the key enzymes of hepatic glucose homeostasis including glycolysis, glycogenesis (glucokinase and glycogen synthase), and gluconeogenesis (pyruvate carboxylase, phosphoenolpyruvate carboxykinase (PEPCK), fructose-1,6-bisphosphatase, and glucose-6-phosphatase), especially in diabetic states. In vitro study showed that ferulic acid (500 µM) and caffeic acid (500 µM) lowered glucose production through suppression of gluconeogenesis and glycogenolysis [59]. Ferulic acid and caffeic acid also increased the level of glucokinase mRNA in rat hepatoma Fao cells [59]. It is assumed that the mechanism of ferulic acid and caffeic acid for reduced hepatic glucose production could be explained by an increase in glucokinase mRNA expression. In addition, caffeic acid (12.5 µM) and cinnamic acid (12.5 µM) have been reported to improve insulin resistance by promoting insulin receptor tyrosyl phosphorylation and up-regulating the expression of insulin signal associated proteins, including insulin receptor, phosphatidylinositol-3 kinase, glycogen synthase, and glucose transporter-2 (GLUT2), leading to enhance the uptake of glucose in tumor necrosis factor-α induced insulin resistant mouse liver FL83B cells [60]. Huang et al. suggest that caffeic acid and cinnamic acid help insulin-resistant FL83B cells improve insulin sensitivity, restore the glucose uptake, and promote glucose utilization as a consequence. They also speculate the concentration of caffeic acid in blood as estimated by calculation from the equation. When compared to the amount of hydroxycinnamic acid intake per day, they suggest that the concentration of caffeic acid in the present study is in the range, which corresponds with its plasma concentration following consumption of coffee, a major source of caffeic acid [60]. Jung et al. demonstrated that supplementation of diabetic mice with caffeic acid (0.02%) lowered the blood glucose and glycosylated hemoglobin levels in diabetic mice strain C57BL/KsJ-db/db through attenuation of hepatic glucose output [61]. The potential mechanisms to explain the amelioration by the supplementation of caffeic acid involved in the increased hepatic glucokinase activity and its mRNA expression and glycogen content accompanied with lowered glucose-6-phosphatase and phosphoenolpyruvate carboxykinase activities and their respective mRNA expressions.

Liu et al. described that a single intravenous injection of isoferulic acid (1–10 mg/kg) reduced the plasma glucose concentration in STZ-diabetic rats [62]. Repeated intravenous injection of isoferulic acid (5 mg/kg), every 8 h, three times per day, reversed the elevated mRNA level of PEPCK in liver of STZ-diabetic rats to the normal level. The mRNA level of GLUT4 in soleus muscle was upregulated by isoferulic acid after repeated treatment. A similar investigation using STZ-induced diabetic models has clearly demonstrated the mechanisms of isoferulic acid on modulation of mRNA expression of PEPCK and GLUT4 [63]. In the insulin deficiency state, isoferulic acid activated α-1A adrenoceptors to enhance the secretion of endogenous β-endorphin from the adrenal gland in STZ-induced diabetic rats [63]. The release of β-endorphin subsequently stimulated the opioid μ-receptors, leading to an increased expression of mRNA GLUT4 in soleus muscle and the suppressed expression of mRNA phosphoenolpyruvate carboxykinase (PEPCK) expression in the liver.

The daily administration of *p*-methoxycinnamic acid (40 mg/kg) for four weeks improved fasting plasma glucose concentration without changes in plasma insulin level in STZ-induced diabetic rats [64]. In addition, *p*-methoxycinnamic acid evidently increased hepatic glycogen storage concomitant with the raised activity of glycolytic enzymes including hexokinase, glucokinase, and phosphofructokinase as well as the suppressed activity of gluconeogenic enzyme such as glucose-6-phosphatase. The alteration of glycolytic and gluconeogenic enzyme activity was not observed in normal rats treated with *p*-methoxycinnamic acid. It was speculated that *p*-methoxycinnamic acid might play a major role in the control of hyperglycemia by decreasing hepatic glucose production in the insulin deficiency state.

Ferulic acid (0.05 g/kg/day) has been shown to effectively suppress blood glucose and increase plasma insulin levels in C57BL/KsJ-db/db diabetic mice by elevating glucokinase activity and production of hepatic glycogen [65]. Narasimhan et al. also confirmed consistent findings of ferulic acid in high-fat diet and fructose-induced type-2 diabetic rats [66]. Ferulic acid treatment (50 mg/kg) to diabetic rats improved blood glucose, serum insulin, glucose tolerance, and insulin tolerance to normal range. The alteration of hepatic glycogen concentration, activity of glycogen synthase, and glucokinase restored to normal level similar to that of metformin after treatment of ferulic acid. The mRNA expression of PEPCK and glucose-6-phosphatase and the interaction between forkhead transcription factor-O1 and promoters of gluconeogenic enzyme genes (PEPCK and glucose-6-phosphatase) was suppressed by ferulic acid indicating the improvement of insulin sensitivity and hepatic glycogenesis in high-fat diet and fructose-induced type-2 diabetic rats. Naowaboot et al. reported the effect of ferulic acid on glucose and lipid metabolism in high-fat diet-induced obese mice [67]. Oral administration of ferulic acid (25 and 50 mg/kg) significantly reduced the elevated blood glucose and lowered the insulin resistance after eight weeks of administration. The reduced expression of hepatic lipogenic genes such as sterol regulatory element-binding protein 1c (SREBP1c), fatty acid synthase (FAS), and acetyl-CoA carboxylase (ACC) concomitant with the up-regulating hepatic carnitine palmitoyltransferase 1a (CPT1a) gene and peroxisome proliferator activated receptor alpha (PPARα) proteins was were observed in the rats treated with ferulic acid. The activation of PPARα, a well-known nuclear receptor, can regulate hepatic lipid metabolism by the reduction of SREBP1c expression and the blockage of the endogenous free fatty acid synthesis via inhibition of ACC and FAS [68]. The activation of PPARα prevents steatosis development and lipid accumulation in the liver [69]. The author concluded that ferulic acid had the beneficial effect on downregulation of genes involved in lipid synthesis and upregulation of genes involved in fat oxidation, leading to reduced lipid accumulation and the prevent of non-alcoholic fatty liver disease (NAFLD). Consistent with the studies by Wang et al. reported that supplementation of 0.05% ferulic acid alleviated high-fat and high-fructose diet-induced obesity, hyperlipidemia, hyperglycemia, hepatic injury, and insulin resistance in rats [70]. In vitro models confirmed that ferulic acid reduced hepatic fat deposition in oleic acid-stimulated HepG2 cells, which was possibly due to the down-regulation of lipogenesis genes expression including FAS, ACCα, ACCβ, and SREBP-2.

Amalan et al. investigated anti-hyperglycemic potential of *p*-hydroxycinnamic acid by evaluating its modulatory effects on the activities of various hepatic enzymes in STZ induced diabetic rats [71]. The orally administered *p*-hydroxycinnamic acid (100 mg/kg) attenuated hyperglycemia and hepatic glucose output via downregulation the expression of liver gluconeogenic enzymes (glucose-6-phosphatase and fructose-1,6-bisphosphatase) and upregulation of glycolytic enzymes (hexokinase, glucose-6 phosphatase dehydrogenase). The effect of *p*-hydroxycinnamic acid on hepatic gluconeogenesis and related pathways was conducted in the perfused rat liver [72]. *p*-Hydroxycinnamic acid markedly inhibited transformation of lactate and alanine into glucose (IC_50_ values of 92.5 μM and 75.6 μM, respectively), whereas glucose 6-phosphatase and fructose 1,6-bisphosphatase (gluconeogenic enzymes) was not inhibited. *p*-Hydroxycinnamic acid also inhibited respiration dependent on pyruvate oxidation, suggesting that the inhibition of pyruvate transport into the mitochondria was the major action of *p*-hydroxycinnamic acid on the suppression of gluconeogenesis in the liver. The effects of cinnamic acid and its derivatives on the hepatic glucose homeostasis are summarized in Table 3.

### 3.6. Adiponectin Secretion

Adiponectin is generally expressed in and secreted from adipose tissue, which plays a vital role in the modulation of glucose and lipid metabolisms in insulin-sensitive tissues such as liver and skeletal muscle [73]. Adiponectin binds its receptors and mediates increased AMPK activities, PPARα activities, fatty-acid oxidation, and glucose uptake, thereby improves insulin sensitivity in muscle and liver. Caffeic acid and ferulic acid (1 μM) have shown the ability to secrete adiponectin in in 3T3-L1 adipocytes [74]. Furthermore, the adiponectin secreting activity of cinnamic acid derivatives was inhibited when NF-κB activation increased [74]. It suggests that caffeic acid and ferulic acid regulate adiponectin secretion through inhibition of NF-κB during inflammatory process. Kopp et al. investigated the mechanisms of cinnamic acid on adiponectin secretion [75]. The authors found that cinnamic acid stimulated adiponectin secretion and increased the phosphorylation of AMPK in 3T3-L1 adipocytes through Gi/Go-protein-coupled receptor signaling pathway. The effects of cinnamic acid and its derivatives on adiponectin secretion are summarized in Table 4.

### 3.7. Adipogenesis

Adipocytes are the main component of adipose tissues and are considered as the regulators of energy balance and glucose homeostasis [76]. Adipogenesis is a multi-step process that requires a cascade of transcription factors including the CCAAT/enhancer-binding protein (C/EBP) gene family and PPARγ that contribute to the development of preadipocytes into mature adipocytes. Adipogenesis includes a two-step developmental process of proliferation and differentiation, which plays critical role on adipose tissue deposition and dysfunction, leading to induce obesity and insulin resistance. The suppressing adipogenesis-related gene expressions such as PPARγ and C/EBPα result in reduced accumulation of triglyceride and thereby improve insulin sensitivity in adipocytes [77]. In the meantime, the activation of PPARγ improves insulin action and glucose uptake by enhancing the insulin signaling pathway in mature adipocytes [78]. As shown by Hsu et al., *o*-hydroxycinnamic acid (50–250 μM) inhibited adipogenesis in 3T3-L1 preadipocytes by decreasing intracellular triglyceride content and inhibiting glycerol-3-phosphate dehydrogenase activity [79]. The inhibitory activity by *o*-hydroxycinnamic acid was mediated through the down-regulated expression of adipogenic transcription factors (PPARγ and C/EBPα and adipocyte-specific proteins (leptin), and then the up-regulated expression of adiponectin [79]. The authors suggest that *o*-hydroxycinnamic acid could be an effective compound for improving adipocyte function during adipogenesis. Furthermore, *p*-hydroxycinnamic acid (10–100 nM) potently suppressed adipogenesis in 3T3-L1 preadipocytes [80]. The suppressive effect of *p*-hydroxycinnamic acid on insulin-stimulated adipogenesis related to the inhibition of the MAPK/ERK signaling pathway in preadipocytes [80]. Cinnamic acid and its derivatives have shown their actions related to adipocyte function. Cinnamic acid (2.5–50 μM) concentration-and time-dependently increased the glucose uptake through PPARγ-mediated GLUT4 translocation in mature 3T3-L1 adipocytes [55]. In addition, cinnamic acid reduced the accumulation of TG and the expression fatty acid synthase and HMG-CoA (5-hydroxy-3-methylglutaryl-coenzyme A) reductase, a rate-limiting enzyme of cholesterol synthesis after differentiation of 3T3-L1 cells. A reduction in fatty acid and cholesterol synthesis might improve insulin resistance as well as the risk of developing type 2 diabetes. The effects of cinnamic acid and its derivatives on inhibition of adipogenesis are summarized in Table 4.

### 3.8. Protein Tyrosine Phosphatase 1B

Protein tyrosine phosphatase 1B (PTP1B) is the enzyme that catalyzes protein tyrosine dephosphorylation from insulin receptor and insulin receptor substrate. The consequence of its action inactivates the downstream insulin signaling cascades and thereby terminates GLUT4 translocation to the plasma membrane, leading to reduce insulin sensitivity [81]. Inhibition of protein tyrosine phosphatase 1B (PTP1B) has been proposed as an attractive target to improve insulin sensitivity in muscle and adipocytes [82]. Several publications have highlighted the inhibitory effect of cinnamic acid and its derivatives against PTP1B. Lakshmi et al. investigated the effect of cinnamic acid isolated from *Cinnamomum cassia* (L.) J.Presl on the inhibition of PTP1B activity [83]. Cinnamic acid (1 nM–0.1 mM) inhibited PTP1B in a concentration-dependent manner. The time course experiments suggested that cinnamic acid was a fast binding inhibitor of PTP1B [83]. In addition, the inhibition of PTP1B by cinnamic acid is reflected by an increase in glucose uptake activity in L6 myotubes. The authors suggest that the inhibition of PTP1B is an alternative mechanism of cinnamic acid to enhance the activation of glucose uptake.

Screening PTP1B inhibitory activity of cinnamic acid and its derivatives has shown the important structure for inhibition of PTP1B [84]. The required key pharmacophore to inhibit PTP1B was the introduction of a hydroxy substituent at the ortho- or para-position on cinnamic acid structure [84]. The studies of enzyme kinetics indicated *o*-hydroxycinnamic acid (IC_50_ = 137.67 μM) and *p*-hydroxycinnamic acid (IC_50_ = 181.6 μM) demonstrated a non-competitive inhibition against PTP1B. The low concentration of *o*-hydroxycinnamic acid and *p*-hydroxycinnamic acid, in combination with a well-known PTP1B inhibitor, sodium orthovanadate, demonstrated a synergistic effect to inhibit PTP1B activity. In vivo studies are necessary to confirm their role in inhibition PTP1B activity. The effects of cinnamic acid and its derivatives on inhibition of protein tyrosine phosphatase 1B are summarized in Table 5.

### 3.9. Pancreatic α-Amylase and α-Glucosidase

α-Glucosidases (α-d-glucoside glucohydrolase) are the carbohydrate digestive enzymes distributed widely in microorganisms, plants, and animal tissues. α-Glucosidases are mainly classified into two families by their substrate recognitions. The α-glucosidase I family consists of the bacterial, yeast and insect enzymes that hydrolyzes the heterogeneous substrates or glucosyl structures, such as *p*-nitrophenyl α-glucoside. The α-glucosidase II family consists of the mold, plants, and mammalian tissues that recognize maltooligosaccharides or maltosyl structures as a substrate [85]. In the human gastrointestinal tract, dietary carbohydrates are digested by salivary α-amylase and followed by pancreatic α-amylase. The products of α-amylase digestion, along with dietary disaccharides are hydrolyzed to their corresponding absorbable monosaccharides in the brush-border surface of intestinal cell by intestinal α-glucosidases such as maltase, sucrase, glucoamylase, and isomaltase. Inhibition of intestinal α-glucosidase aggressively delays the digestion of starch and disaccharides to absorbable monosaccharides, which suppresses a rise on postprandial hyperglycemia. This has been proved to be one of the effective strategies to decrease the postprandial rise in blood glucose and in turn help preventing the onset of diabetes and its complications [86].

A series of cinnamic acid and its derivatives was investigated for their potentials as an inhibitor of yeast [87] and rat intestinal α-glucosidase [88]. Among 17 compounds screened for the inhibitory activity, cinnamic acid was found to be inactive with respect to yeast α-glucosidases. Addition of hydroxy or methoxy group on the structure of cinnamic acid resulted in increased α-glucosidase inhibitory activity. It was found that the presence of methoxy group had a higher potency than hydroxyl group. The observation revealed that *p*-methoxycinnamic acid demonstrated the highest inhibitory activity against yeast α-glucosidase [87]. In contrast to another report in the mammalian enzymes, *p*-methoxycinnamic acid was an inactive inhibitor against intestinal α-glucosidase (sucrase and maltase) [88]. The presence of methoxy group in cinnamic acid resulted in a significant loss of intestinal maltase and sucrase inhibitory activity. Surprisingly, the introduction of hydroxyl group enhanced an increase in intestinal maltase and sucrase inhibitory activity [88]. The authors suggest that the different substrate recognitions and the catalytic regions may directly affect the binding affinity of cinnamic acid derivatives on the different families of α-glucosidases. Furthermore, the addition of two hydroxyl substituents at meta- and para-positions in the structure of cinnamic acid (caffeic acid, isoferulic acid, and ferulic acid) improved α-glucosidase inhibitory activity against intestinal sucrase and maltase [88]. The type of inhibition of caffeic acid, ferulic acid, and isoferulic acid against intestinal maltase was a mixed-inhibition manner (IC_50_ values = 0.74 mM, 0.79 mM, and 0.76 mM, respectively). Ferulic acid (IC_50_ value = 0.45 mM) and isoferulic acid (IC_50_ value = 0.45 mM) displayed mixed noncompetitive mode of inhibition towards intestinal sucrase, whereas caffeic acid (IC_50_ value = 0.49 mM) was a non-competitive inhibition [88]. The combined concentration between acarbose and cinnamic acids derivatives on the inhibition of α-glucosidase has been noted in this study [88]. Low concentration of caffeic acid, isoferulic acid, and ferulic acid could produce additive intestinal sucrase inhibition when combination with low concentration of acarbose. Boath et al. suggest that phytochemical compounds with high α-glucosidase and lower pancreatic α-amylase inhibitory activity could prevent certain side effects of acarbose [89]. The decreasing required dose of acarbose by combined with phytochemical compounds might diminish its gastrointestinal side effects such as flatulence, diarrhea and abdominal distension [90].

The interaction of caffeic acid and two active subunits located on the respective C and N terminals of human α-glucosidases maltase-glucoamylase and sucrase-isomaltase has been described [91]. Simsek et al. proposed that the highly active C terminal-subunits of α-glucosidases are a vital binding site for the inhibition of carbohydrate digestion. In this regard, caffeic acid demonstrated higher binding affinities for the C terminal-subunits than the N-terminal subunits of maltase-glucoamylase and sucrase-isomaltase [91]. This suggests that the binding causes the slowdown of starch digestion and reduction of the glycemic spike. The interaction between caffeic acid and the aromatic motifs in proximity to the active site leads to impair the binding of the substrate with a correct orientation, which has been speculated for the inhibitory mechanism of caffeic acid [92]. Attempts to the structural modification of caffeic acid have become an alternative to improve the intestinal α-glucosidase inhibitory activity by mimicking the corresponding natural substrates. With successful synthesized bioconjugation, coupling of caffeic acid with quercitol had 6 times greater inhibitory potency toward maltase than its parent compounds [93]. Addition of sugar-amides (aminomethyl-β-d-glucopyranoside moiety) into cinnamic acid derivatives led to an increased potency against intestinal maltase with equivalent potency as acarbose [94].

Narita et al. described the inhibitory effects of cinnamic acid derivatives on porcine α-amylase [95]. It was observed that the inhibitory activities of cinnamic acid derivatives were in the order of caffeic acid > isoferulic acid > *m*-hydroxycinnamic acid = *p*-hydroxycinnamic acid > ferulic acid > *m*-methoxycinnamic acid = *p*-methoxycinnamic acid. Introduction of the hydroxyl group enhanced the pancreatic α-amylase inhibitory effects, whereas it was minor affected by the presence of the methoxy group in the structure of cinnamic acid. There are some structural features of cinnamic acid considered for the inhibition on porcine α-amylase, which became the strongest with the introduction of hydroxylation at para- and meta-position of cinnamic acid structure [95]. The effects of cinnamic acid and its derivatives on inhibition of intestinal α-glucosidase and porcine α-amylase are summarized in Table 5.

### 3.10. Intestinal Glucose Absorption

**Figure 4:**
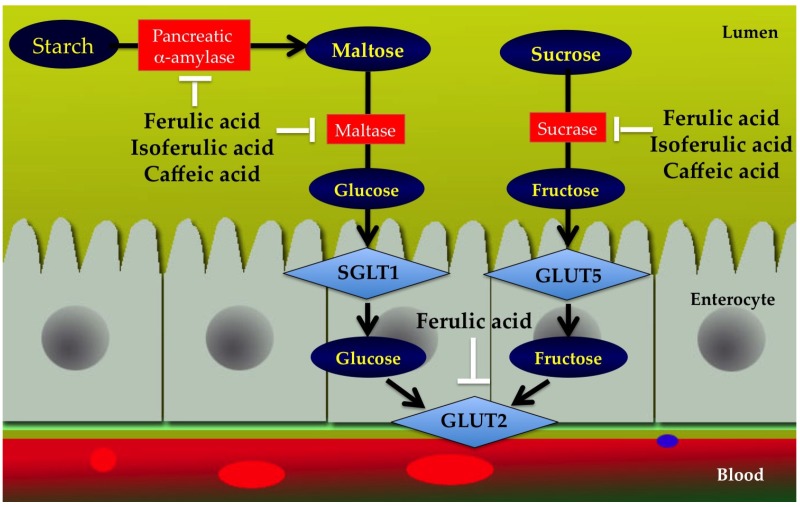
Inhibition of carbohydrate digestive enzymes (pancreatic α-amylase, maltase, and sucrase) and monosaccharide absorption (GLUT2) by ferulic acid, isoferulic acid, and caffeic acid.

Inhibition of transporters responsible for dietary monosaccharides glucose from the intestinal tract could have substantial impact in controlling postprandial hyperglycemia [96]. Dietary glucose derived either from hydrolysis of starch or from sucrose is mainly taken up into the apical enterocyte membrane by the sodium-dependent glucose co-transporter-1 (SGLT1) and facilitated glucose transport (GLUT5) from enterocytes into blood mediated by glucose transporter 2 (GLUT2). Malunga et al. reported that ferulic acid markedly inhibited intestinal glucose uptake by interfering GLUT2 [97]. Likewise, ferulic acid moiety of feruloyl arabinoxylan mono/oligosaccharide, the simplest compound present naturally in cereal grains could also inhibit glucose absorption through GLUT2. These observations suggest that ferulic acid moiety might be the active site for inhibiting glucose absorption. Inhibition of carbohydrate digestive enzymes and monosaccharide absorption by ferulic acid, isoferulic acid, and caffeic acid are shown in Figure 4.

Whole-grain intake has been remarkably associated with a reduction in the risk of type 2 diabetes [98,99]. Evidence from clinical trials in humans has indicated that consumption of whole grain diet results in a lower blood glucose concentration [100]. The major bioactive compounds responsible for lowering blood glucose effect of whole grain might be in part explained by the presence of ferulic acid and feruloyl mono/oligosaccharides. Other cinnamic acid derivatives including caffeic acid and *p*-hydroxycinnamic acid had no effect on inhibition of glucose uptake under either sodium-dependent or sodium-free conditions [101]. The effects of cinnamic acid and its derivatives on inhibition of glucose absorption are summarized in Table 5.

### 3.11. Protein Glycation

Long-term hyperglycemia is a major causative factor to induce non-enzymatic protein glycation by reducing sugars, such as glucose and fructose, which react with the free amino groups of proteins [102]. The reaction produces an unstable Schiff base, and then forms an Amadori product such a fructosamine. During the propagation reaction, the Amadori products react with the amino acids to form the irreversible advanced glycation end products (AGEs). The accumulation of AGEs in human tissues has been associated with development of several age-related degeneration, atherosclerosis and diabetic complications such as retinopathy, nephropathy and neuropathy [102]. There has been interest in finding effective agents with anti-glycation activity, which is a therapeutic strategy for prevention and amelioration of AGE-mediated diabetic complications [103].

Several reports regarding anti-glycation activity of cinnamic acid and its derivatives have been published. Anti-glycation effect of cinnamic acid and its derivatives has been evaluated in fructose-induced protein glycation in bovine serum albumin [104]. At the concentration of 1 mM, cinnamic acid demonstrated the strongest AGE inhibitor among seven cinnamic acid derivatives tested. The percentage AGE inhibition of cinnamic acid was the same range comparable to that of aminoguanidine on the weight concentration basis [104]. In addition, the introduction of a hydroxyl group or a methoxy group on the structure of cinnamic acid was decreased the abilities to inhibit the formation of AGEs. The reduced level of fructosamine (Amadori products) and non-fluorescent AGEs (Nε-(carboxymethyl) lysine (CML)) by cinnamic acid and its derivatives was also observed in this study. The findings suggest that cinnamic acid and its derivatives are effective in reducing CML formation associated with reduced levels of fructosamine. The consistent results obtained by Qais et al. indicated that cinnamic acid (25–200 μM) prevented glucose-induced AGE formation in human serum albumin (HSA) [105].

In presence of two substituents in cinnamic acid structure, ferulic acid (1–5 mM) has been shown to protect against glucose-, fructose- and ribose-induced formation of fluorescent AGEs in BSA [106]. The glycating ability of monosaccharides occurred in the following increasing order: ribose > fructose > glucose. Comparing at the same concentration, ferulic acid had greater percentage inhibition in fructose-glycated BSA than glucose-glycated BSA [106]. In a similar study, an isomer of ferulic acid, isoferulic acid (1.25–5 mM), inhibited the formation of fluorescent AGEs and non-fluorescent AGE (N^δ^-CML) and fructosamine protein adducts in glucose- and fructose-induced protein glycation [107]. Taken together, the position of methoxy and hydroxy group on ferulic acid and isoferulic acid have no effect on the ability to inhibit protein glycation. In fructose and glucose model, caffeic acid exhibited inhibitory activity against the formation of AGEs [108]. Protein glycation accelerates the formation of protein aggregation and amyloid cross β-structures leading to altered protein structure, stability, and function. Elevated levels of protein aggregation cause neurodegeneration in patients with Alzheimer’s disease [109]. The accumulation of amyloid cross β-structures induces pancreatic islet amyloidosis that results in β-cell damage and impaired insulin secretion in diabetic patients [110]. Ferulic acid (1–5 mM) and isoferulic acid (1.25–5 mM) could effectively reduce the formation of amyloid cross β-structures in glucose- and fructose-glycated BSA. Inhibition of formation of amyloid cross β-structures by these compounds may help reduce the progression of pancreatic islet amyloidosis.

Molecular docking studies further characterized the interaction between cinnamic acid and its derivatives by using albumin. The inhibitory effect of cinnamic acid on AGE formation may be caused by its binding on HSA subdomain IIA (Sudlow’s site I) [105]. Cinnamic acid forms two hydrogen bonds with Ser287 and Arg257 and hydrophobic interactions with Leu219, Leu234, Leu238, Ile264 and Leu260 of HSA. The interaction leads to stabilize the HSA-Cinnamic acid complex, and reduced numbers of glycating sites with free glucose, thereby inhibiting protein glycation. In another study, molecular docking was performed to provide better understanding of the binding interaction between cinnamic acid derivatives and bovine serum albumin [111]. The binding affinity ranked in the order caffeic acid > *m*-hydroxycinnamic acid ≥ *p*-hydroxycinnamic acid > ferulic acid. The authors suggest that introduction of two hydroxyl groups in the structure of cinnamic acid (caffeic acid) leads to higher binding ability compared to that of the presence of only one hydroxyl group (*m*-hydroxycinnamic acid and *p*-hydroxycinnamic acid). In contrast, the affinity of ferulic acid to albumin was observed much smaller because of the steric hindrance of methoxy group on the structure. Consistent with the binding of HSA with cinnamic acid, caffeic acid, *m*-hydroxycinnamic acid, *p*-hydroxycinnamic acid, and ferulic acid also bind to site I in the subdomain IIA of BSA through the hydrophobic interaction and hydrogen bonding. Molecular docking revealed that the phenyl group in the structure of caffeic acid, *m*-hydroxycinnamic acid, and *p*-hydroxycinnamic acid is the region for interaction with BSA binding socket.

**Figure 5:**
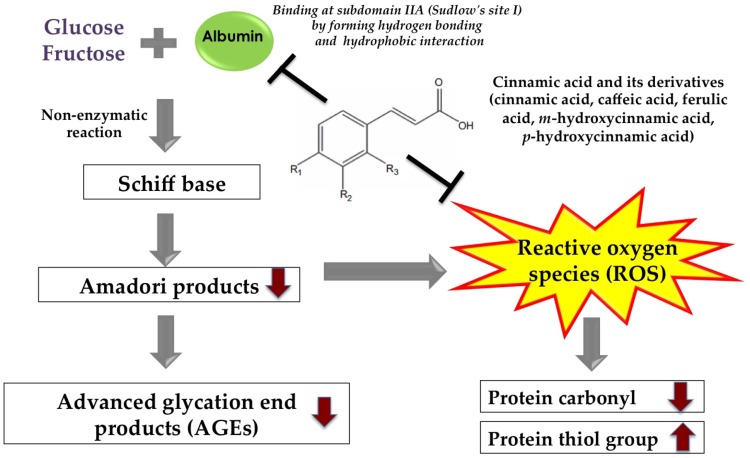
Graphical presentation of possible anti-glycation mechanisms of cinnamic acid and its derivatives.

Glycation is not only an important cause of AGE-induced protein modification and alteration, but it also induces oxidation-dependent tissues and protein damage. During the glycation process, the oxidative degradation of Amadori intermediates generates superoxide anion such as 1,2- and 2,3-enolization of the Schiff’s base causing oxidative damage to protein detected by increasing formation of protein carbonyl and depleting protein thiol group [112]. It was shown that the presence of cinnamic acid (1 mM), hydroxycinnamic acids (1 mM) and methoxycinnamic acids (1 mM) reduced the formation of protein carbonyl and the depleting protein thiol group when incubated with bovine serum albumin and fructose [107]. Most cinnamic acid and its derivatives have been known as potent antioxidant phytochemicals due to their unique structure [113]. It is described that the mechanism responsible for inhibition of glycation-induced protein oxidation by cinnamic acid and its derivatives may be related to their antioxidant activity. The possible mechanisms underlying the anti-glycation activity of cinnamic acid and its derivatives are represented in Figure 5.

Glycated hemoglobin (Amadori products) is also produced from non-enzymatic glycation between glucose and N-terminal amino acids of beta chains of hemoglobin. Higher glycated hemoglobin level is mainly attributed to modulatory effects on dysfunction of membrane ion pumps (Na^+^/K^+^-ATPase) pump and the formation of membrane lipid peroxidation [114]. Altered membrane ion pumps are associated with increased risk of developing diabetic vascular complications [115]. Treatment with ferulic acid reduced high glucose-induced glycated hemoglobin A1C (HbA1c) [116]. It suggests that reduced formation of HbA1c may be due to increased glucose utilization, thereby decreasing intracellular glucose [116]. There was evidence indicating that ferulic acid (0.1–100 μM) improved hyperglycemia-induced impairment of Na^+^/K^+^-ATPase activity and reduced the formation of lipid peroxidation in glycated hemoglobin.

**Figure 6:**
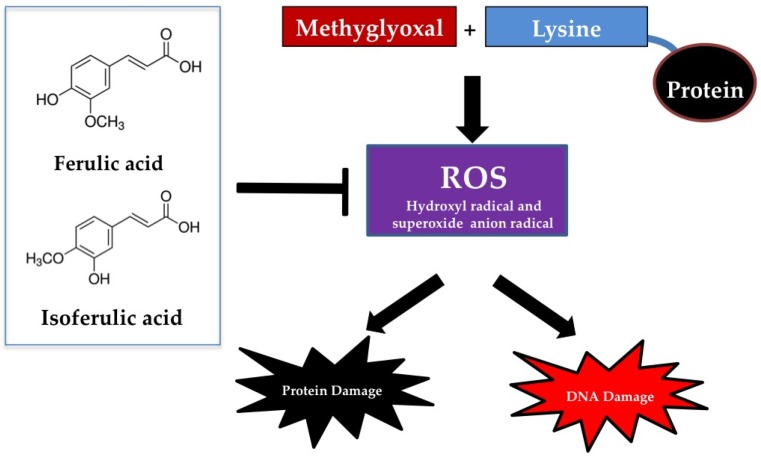
The mechanism of ferulic acid and isoferulic acid on prevention of methylglyoxal-induced protein and DNA damage.

Methylglyoxal, a highly reactive agent, is formed during the metabolic intermediates of carbohydrates, fatty acids, and proteins [117]. Methylglyoxal rapidly binds to amino acids such as lysine, arginine, and cysteine residues to form AGEs in the late stage of protein glycation [118]. Besides protein glycation, methylglyoxal also induces oxidative DNA damage and cell cytotoxicity, resulting in conditions like cell apoptosis [119]. Evidence supports that reactive oxygen species (ROS) are generated from the reaction of amino acid (lysine) with methylglyoxal [119]. Several works have shown the effects of cinnamic acid derivatives on methylglyoxal-induced protein glycation. For example, ferulic acid (0.1–1 mM) has been described as able to reduce methylglyoxal-induced formation of AGEs and oxidative damage to BSA [120]. The similar effect was obtained by incubation of isoferulic acid together with methylglyoxal and BSA [121]. Isoferulic acid (1.25–5 mM) markedly inhibited methylglyoxal-induced formation of AGEs and oxidative protein damage in BSA [121]. Both ferulic acid (0.0125–0.2 mM) and isoferulic acid (0.1–1 mM) effectively prevented oxidative DNA damage during the reaction of lysine and methylglyoxal [120,121]. The protective ability of ferulic acid and isoferulic acid was directly correlated to the inhibition of hydroxyl and superoxide anion radical generation during the reaction of MG and lysine. Other mechanisms related to the ability to trap methylglyoxal have been proposed but it is clearly shown that ferulic acid and isoferulic acid did not directly react with methylglyoxal [120,121]. The mechanism of ferulic acid and isoferulic acid responsible for the inhibition of methylglyoxal-induced protein glycation and DNA damage is free radical scavenging activity without the MG-trapping ability (Figure 6). Caffeic acid, a bioactive substance isolated from yerba maté (*Ilex paraguariensis* A.St.-Hil.), demonstrated anti-glycation effect on methylglyoxal-induced protein glycation in BSA and histone [122]. Crosslink structures of BSA and histone produced by methylglyoxal were significantly blocked by caffeic acid. Furthermore, *p*-hydroxycinnamic acid was reported to slightly inhibit protein glycation induced by methylglyoxal and glyoxal [123].

Besides the effect of methylglyoxal-induced protein glycation in albumin, the preventive effect of isoferulic acid has been reported in glycation-mediated oxidation of human HDL [124]. Isoferulic acid prevented methylglyoxal-induced changes in structural and functional properties of human HDL by improving number of free amino group, thermal denaturing profiles, and paraoxonase activity. This suggests that the effect of isofeulic acid could also protect the loss of anti-inflammatory and antioxidant activity of HDL resulting in prevention of diabetes-associated cardiovascular diseases.

Some protective effects of cinnamic acid and its derivatives have demonstrated the ability to modulate the function and survival in various cells. Pretreatment of ferulic acid (100 μM) followed by methylglyoxal (1 mM) attenuated the cell cytotoxicity in INS-1 pancreatic β-cells [120]. It restored the cell survival through the suppression of methylglyoxal-mediated cell apoptosis. There is a report demonstrating the potential benefits of cinnamic acid and its derivatives against methylglyoxal and glyoxal-induced cytotoxicity and oxidative stress in hepatocytes [125]. The order of protection was caffeic acid = ferulic acid > ferulaldehyde > ethyl ferulate = methyl ferulate > *p*-hydroxycinnamic acid. Ferulic acid significantly reduced glyoxal- or methylglyoxal-induced cytotoxicity, and ROS formation as well as improved mitochondrial membrane potential in various models in depleting antioxidant systems and inflammation of hepatocytes [125]. By the same mechanism, ferulic acid acts as a free radical scavenger against methylglyoxal-induced oxidative stress in hepatocytes. The effects of cinnamic acid and its derivatives on inhibition of protein glycation are demonstrated in Table 6.

### 3.12. Insulin Fibrillation

Insulin fibrillation, characterized by β-sheet rich structure, is present in arterial walls and on membrane surfaces [126]. It has been shown that aggregation and fibrillation of insulin are also produced during production, storage, and delivery of insulin based drugs [127]. In addition, insulin amyloid fibrils were also found in type 2 diabetic patients after a single or repeated subcutaneous injection of insulin [128]. There was an interesting data demonstrating that insulin fibrillation induced cytotoxicity in the pheochromocytoma PC12 and human neuroblastoma SH-SY5Y cell lines [129]. Moreover, insulin fibrils induced the aggregation of cytoskeletal proteins of erythrocyte membranes, causing the hemolysis in erythrocytes [130]. Jayamani et al. demonstrated the inhibitory effect of ferulic acid on insulin amyloid fibril formation. The minimum molar ratio of insulin:ferulic acid (1:10) displayed the maximum inhibition of insulin fibrillation approximately 85% [131]. Ferulic acid prevented the conformational transition from α-helix to β-sheet. Molecular docking analysis revealed that the phenyl, hydroxyl and carboxylic moieties of ferulic acid interact with insulin through hydrogen bonds as well as hydrophobic interactions [131]. It is proposed that ferulic acid can be considered for delaying the growth of amyloidosis.

## 4. New Formulation of Cinnamic Acid and Its Derivatives

Data on the bioavailability and other pharmacokinetic properties of cinnamic acid and its derivatives reveal that low plasma concentrations are most likely due to limited absorption, intensive metabolism and/or fast elimination of cinnamic acid and its derivatives from circulation [132]. These effects may not be sufficient to produce significant in vivo biological effects needed for prevention of chronic diseases. In order to increase cinnamic acid and its derivatives bioavailability and enhance its biological effects, new formulations have been prepared in which cinnamic acid derivatives are entrapped into solid and liquid particles. For example, self-nanoemulsifying drug delivery system (SNEDDS) of cinnamic acid was developed in order to increase its bioavailability and anti-diabetic action [133]. The bioavailability study revealed that the C_max_ of cinnamic acid SNEDDS had 2.5-fold higher than that of cinnamic acid suspension *in the rat model*. However, cinnamic acid SNEDDS had the same efficacy to reduce blood glucose and cholesterol level in alloxan-induced diabetic rats when compared to cinnamic acid suspension. In another study, liquid anti-solvent precipitate process was also employed to prepare the cinnamic acid nanoparticle [134]. This formulation had 2.16-fold higher oral bioavailability than that of unencapsulated cinnamic acid. In another study, a microemulsion (ME)-based technique was used to prepare nanostructured lipid carriers (NLCs) and solid lipid nanoparticles (SLNs) as vehicles for ferulic acid [135]. After oral administration of different types of ferulic acid-loaded particles (80 mg/kg) to the rats, SLNs and NLCs had a greater oral bioavailability of ferulic acid than non-encapsulated ferulic acid. Both ferulic acid-NLCs (t_1/2_ = 4.69 h) and SLNs (t_1/2_ = 4.59 h) had longer biological half-life than ferulic acid-unloaded nanoparticles (t_1/2_ = 2.14 h). In addition, ferulic acid was encapsulated within amaranth protein isolate (API) and pullulan ultrathin fibers using the electrospinning technique [136]. The ferulic acid encapsulation improved antioxidant capacity in comparison with free ferulic under simulated gastrointestinal digestion. These formulations might be a useful platform for the possible translation to clinical evaluation.

## 5. Conclusions and Perspectives

Several mechanisms have been proposed to explain the effect of cinnamic acid and its derivatives on prevention and management of diabetes and its complications (Table 1). Even though the potential benefits of cinnamic acid and its derivatives have been demonstrated in the in vitro and preclinical studies, there is no clinical evidence to prove the beneficial effects of cinnamic acid and its derivatives. Furthermore, further epidemiological studies are required to evaluate the role of cinnamic acid and its derivatives in prevention and management of diabetes and its complications. The main concerns derive from the pharmacokinetics of these compounds, in particular, low bioavailability; thus, the use of cinnamic acid and its derivatives in the field of pharmaceuticals and nutraceuticals is still limited. Current efforts to improve bioavailability of cinnamic acid and its derivatives have successfully developed novel applications, including nanoparticles, encapsulation, and emulsions. In the future perspective of research, additional approach of clinical studies may help us to understand the full potential of cinnamic acid and its derivatives in prevention and management of diabetes and its complications.

## Figures and Tables

**Table 1:** A summary of the mechanisms through which cinnamic acid and its derivatives could stimulate insulin secretion and improve pancreatic β-cell functionality.

Mechanisms	Effects
Insulin secretion	*m-*Hydroxycinnamic acid, *p-*methoxycinnamic acid, and ferulic acid stimulate insulin secretion from INS-1 pancreatic β-cells and the pancreatic rat perfusion technique
	Ferulic acid reduces plasma glucose and increases plasma insulin level in normal rats
	*p-*Methoxycinnamic acid stimulates insulin secretion through activation of l-type Ca^2+^ channel
	*p-*Methoxycinnamic acid reduces plasma glucose and increases plasma insulin level in normal and STZ-induced diabetic rats
	Cinnamic acid enhances glucose-induced insulin secretion in the isolated mice islets
	Cinnamic acid improves glucose tolerance in diabetic rats
	Caffeic acid enhances glucose-induced insulin secretion in INS-1 pancreatic β-cells
Pancreatic β-cell functionality	Ferulic acid regenerates pancreatic islets in STZ-induced diabetic rats and reduces apoptosis and inflammation through a decline in level of IL-1β and TGF-β1
	Ferulic acid increases islet number and sizes and reduced insulitis grades in diabetic rats when co-administration with metformin and thiazolidinedione
	Cinnamic acid prevents palmitic acid-induced lipotoxicity in mouse NIT-1 pancreatic β-cells
	Cinnamic acid inhibits palmitic acid-induced alteration of lipogenic gene and protein expression (AMPK, SREBP-1c, FAS, ACC)

**Table 2:** A summary of the mechanisms through which cinnamic acid and its derivatives could inhibit dipeptidyl peptidase-IV and stimulate the glucose uptake.

Mechanisms	Effects
Dipeptidyl peptidase-IV	Cinnamic acid, *p*-methoxycinnamic acid, and caffeic acid showed inhibitory activity against DPPIV
	Hydrogen bonding and the formation of π-interaction are the main binding mode of caffeic acid with DPP-IV
Glucose uptake	Isoferulic acid activates α-1A adrenoceptor, thereby stimulating glucose uptake via PLC-PKC pathway in C_2_C_12_ cells
	Ferulic acid, caffeic acid, and *p*-hydroxycinnamic acid stimulate the glucose uptake mediated by PI3K-dependent GLUT4 translocation, whereas cinnamic acid increases the glucose uptake via PPARγ-mediated GLUT4 translocation in mature 3T3-L1 adipocytes
	Ferulic acid, caffeic acid, and *p*-hydroxycinnamic acid synergistically interacted with metformin and thiazolidinedione, whereas cinnamic acid exhibited an additive effect on the uptake of glucose
	Combination of ferulic acid with metformin or thiazolidinedione demonstrates synergistic effect on glucose uptake in rat L6 myotubes
	Ferulic acid prevents saturated fatty acid-induced defects in the insulin receptor through the blockage of PKCε activation and thereby permission of HMGA1 to activate insulin receptor β promoter in rat L6 myotubes

**Table 3:** A summary of the mechanisms through which cinnamic acid and its derivatives could modulate hepatic glucose homeostasis.

Mechanisms	Effects
Hepatic glucose homeostasis	Ferulic acid and caffeic acid lower glucose production through suppression of gluconeogenesis and glycogenolysis by increasing the level of glucokinase mRNA in rat hepatoma Fao cells
	Caffeic acid and cinnamic acid improve insulin resistance in tumor necrosis factor-α induced insulin resistant mouse liver FL83B cells by promoting insulin receptor tyrosyl phosphorylation and up-regulating the expression of insulin signal associated proteins, including insulin receptor, phosphatidylinositol-3 kinase, glycogen synthase, and glucose transporter-2 (GLUT2)
	Caffeic acid lowers the blood glucose and glycosylated hemoglobin levels in diabetic mice strain C57BL/KsJ-db/db by an attenuation of hepatic glucose output
	Isoferulic acid modulates mRNA expression of PEPCK and GLUT4 in STZ-induced diabetic rats by activating α-1A adrenoceptors to enhance the secretion of endogenous β-endorphin
	*p*-Methoxycinnamic acid decreases plasma glucose level in STZ-diabetic rats by increased hepatic glycogen storage concomitant with the raised activity of hexokinase, glucokinase and phosphofructokinase and suppressed glucose-6-phosphatase
	Ferulic acid reduces hepatic glucose production by increasing glucokinase mRNA expression
	Ferulic acid suppresses blood glucose in C57BL/KsJ-db/db diabetic mice by elevating glucokinase activity and production of hepatic glycogen increased plasma insulin levels
	Ferulic acid improves blood glucose, serum insulin and glucose tolerance in high-fat diet and fructose-induced type 2 diabetic rats by suppressing mRNA expression of PEPCK and glucose-6-phosphatase
	Ferulic acid reduces the elevated blood glucose and lowers the insulin resistance in high-fat diet-induced obese mice by reducing expression of hepatic SREBP1c, FAS, and ACC concomitant with the up-regulating CPT1a and PPARα
	Ferulic acid reduces hepatic fat deposition in oleic acid-stimulated HepG2 cells by the down-regulation of gene expression of FAS, ACCα, ACCβ, and SREBP-2
	*p*-Hydroxycinnamic acid suppresses gluconeogenesis the rat perfused liver by inhibiting transformation of lactate and alanine into glucose

**Table 4:** A summary of the mechanisms through which cinnamic acid and its derivatives could stimulate adiponectin secretion and inhibit adipogenesis.

Mechanisms	Effects
Adiponectin secretion	Caffeic acid and ferulic acid regulate adiponectin secretion through inhibition of NF-κB during inflammatory process
	Cinnamic acid stimulates adiponectin secretion and increased the phosphorylation of AMPK in 3T3-L1 adipocytes through Gi/Go-protein-coupled receptor signaling pathway
Adipogenesis	*o*-Hydroxycinnamic acid decreases intracellular triglyceride content and inhibits glycerol-3-phosphate dehydrogenase activity in 3T3-L1 preadipocytes through the down-regulated expression of adipogenic transcription factors (PPARγ and C/EBPα and adipocyte-specific proteins (leptin)
	*p-*Hydroxycinnamic acid suppresses adipogenesis in 3T3-L1 preadipocytes through the inhibition of the MAPK/ERK signaling pathway in preadipocytes

**Table 5:** A summary of the mechanisms through which cinnamic acid and its derivatives could inhibit protein tyrosine phosphatase 1B, intestinal α-glucosidase, pancreatic α-amylase, and glucose absorption.

Mechanisms	Effects
Protein tyrosine phosphatase 1B	Cinnamic acid inhibits PTP1B with a fast binding
	*o*-Hydroxycinnamic acid and *p*-hydroxycinnamic acid demonstrate a non-competitive inhibition against PTP1B
Pancreatic α-amylase and α-glucosidase	Caffeic acid, ferulic acid, and isoferulic acid demonstrate a mixed-inhibition against intestinal maltase
	Ferulic acid and isoferulic acid display mixed noncompetitive mode of inhibition towards intestinal sucrase, whereas caffeic acid is a non-competitive inhibition
	Caffeic acid, isoferulic acid, *m*-hydroxycinnamic acid, ferulic acid, *p*-hydroxycinnamic acid, *m*-methoxycinnamic acid, and *p*-methoxycinnamic acid inhibit pancreatic α-amylase
Intestinal glucose absorption	Ferulic acid inhibits intestinal glucose uptake by interfering GLUT2

**Table 6:** A summary of the mechanisms through which cinnamic acid and its derivatives could inhibit protein glycation and insulin fibrillation.

Mechanisms	Effects
Protein glycation	Cinnamic acid inhibits fructose-induced AGEs and fructosamine formation in bovine serum albumin
	Cinnamic acid inhibits glucose-induced protein glycation in human serum albumin
	*p*-Hydroxycinnamic acid inhibits methylglyoxal- and glyoxal-induced protein glycation in bovine serum albumin
	Ferulic acid inhibits glucose-, fructose-, and ribose-induced AGEs and fructosamine formation in bovine serum albumin
	Ferulic acid and isoferulic acid prevent glycation-induced oxidative damage to bovine serum albumin
	Ferulic acid reduces high glucose-induced glycated hemoglobin, lipid peroxidation, and impairment of Na^+^/K^+^-ATPase activity
	Ferulic acid and isoferulic acid inhibit methylglyoxal-induced protein glycation and oxidative damage to protein and DNA
	Ferulic acid prevents methylglyoxal-mediated cell apoptosis and oxidative stress in HepG2 cells
	Ferulic acid prevents methylglyoxal-mediated cell apoptosis in pancreatic β-cells
	Isoferulic acid prevents methylglyoxal-induced changes in structural and functional properties of human HDL
	Caffeic acid inhibits glucose- and fructose-induced AGEs and fructosamine formation in bovine serum
	Caffeic acid inhibits methylglyoxal-induced protein glycation in bovine serum albumin and histone
Insulin fibrillation	Ferulic acid inhibits the formation of insulin amyloid fibril

## Abbreviations

ACC	acetyl-CoA carboxylase
AGEs	advanced glycation end products
AMPK	adenosine monophosphate-activated protein kinase
BSA	bovine serum albumin
C/EBP	CCAAT/enhancer-binding protein
CPT1a	hepatic carnitine palmitoyltransferase 1a
DPP-4	dipeptidyl peptidase-4
FAS	fatty acid synthase
GLUT	glucose transporter
HSA	human serum albumin
PPARα	peroxisome proliferator activated receptor alpha
PPARγ	peroxisome proliferator activated receptor gamma
PTP1B	protein tyrosine phosphatase 1B
SGLT-1	sodium-dependent glucose co-transporter-1
STZ	streptozotocin
